# Advancements in CRISPR/Cas Technologies for Sensitive Cancer Detection: Mechanisms, Platforms, and Clinical Translation Roadmap

**DOI:** 10.3390/diagnostics16162531

**Published:** 2026-08-11

**Authors:** Rokeya Akter, Sook Won Ryu, Jong-Han Lee

**Affiliations:** 1 Department of Laboratory Medicine, Wonju College of Medicine, Yonsei University, Wonju 26426, Republic of Korea; akterbinteha30@yonsei.ac.kr; 2 Department of Laboratory Medicine, Kangwon National University School of Medicine, Chuncheon 24341, Republic of Korea; ryusw@kangwon.ac.kr; 3 Research Institute of Metabolism and Inflammation, Wonju College of Medicine, Yonsei University, Wonju 26426, Republic of Korea

**Keywords:** CRISPR/Cas diagnostics (CRISPR/Cas-Dx), liquid biopsy, early-stage cancer detection, multi-omics biomarkers, digital CRISPR, AI-assisted diagnostics, clinical translation

## Abstract

Early cancer detection is critical for improving patient outcomes, yet current diagnostic approaches often fail to identify malignancies when tumor-specific biomarkers are relatively scarce. Emerging CRISPR/Cas-based technologies have revolutionized the ultra-sensitive detection of cancer biomarkers in liquid biopsies, overcoming the inherent limitations of traditional diagnostic approaches such as tissue biopsies and imaging, which frequently fail to detect early-stage malignancies with sufficient sensitivity. This review explores recent advances in CRISPR/Cas diagnostics (CRISPR/Cas-Dx) that employ programmable CRISPR effectors, including Cas9, Cas12, Cas13, and Cas14. In particular, the collateral (trans-) cleavage activities of Cas12, Cas13, and Cas14 enable signal amplification for highly sensitive detection of circulating tumor DNA, microRNAs, exosomes, and other multi-omics biomarkers, often without the need for extensive nucleic acid amplification. Representative CRISPR/Cas-Dx platforms include amplification-coupled assays, amplification-free frameworks, biosensing and multiplexing capabilities, and new digital or droplet-based configurations that include artificial intelligence to improve analytical precision. These technologies demonstrate single-molecule resolution and adaptability for point-of-care testing in malignancies such as non-small cell lung, colorectal, and breast carcinoma. Finally, we outline a clinical translation roadmap encompassing manufacturability, regulatory and standardization requirements, and real-world implementation challenges. This perspective offers a blueprint for CRISPR-powered, ultra-sensitive liquid biopsy diagnostics that can enable population-scale early cancer screening and truly preventive oncology by bridging molecular insights, engineering innovation, and clinical imperatives.

## 1. Introduction

One of the most effective ways to improve clinical results for cancer patients is still early cancer identification. Patients who receive an early stage or presymptomatic diagnosis frequently experience significantly higher survival rates, less invasive treatments, and reduced toxicity. Traditional diagnostic modalities including radiological imaging such as magnetic resonance imaging (MRI), computed tomography (CT), positron emission tomography (PET), as well as tissue biopsies, remain the clinical standard, yet they suffer from important limitations [1,2]. Tissue biopsies are invasive, frequently impractical for repeated monitoring, and prone to sample bias, while imaging typically detects cancers only after they have grown to a certain size [3]. Although useful, protein biomarkers like CA 125, prostate specific antigen (PSA), or carcinoembryonic antigen (CEA) lack adequate sensitivity and specificity in many early stage disease situations, particularly when tumor burden is low [4]. Liquid biopsy identifies circulating tumor DNA (ctDNA), circulating cell-free RNA (cfRNA), extracellular vesicles (EVs), microRNAs (miRNAs), circulating tumor cells (CTCs), and other tumor-derived biomarkers before radiological or clinical indications appear. Early-stage scarcity presents hurdles, as non-tumor vesicles hide exosomal miRNAs, and ctDNA may constitute only a very small fraction of total cfDNA, particularly in early-stage disease. Detection requires ultrasensitive, highly selective platforms [5,6,7]. To address this challenge, CRISPR/Cas-Dx have emerged quickly. Clustered regularly interspaced short palindromic repeats (CRISPR) systems, which were first developed for genome editing, have been repurposed for molecular diagnostics due to their remarkable precision in recognizing almost any nucleic acid sequence [8]. Because they can be integrated with isothermal amplification methods, can withstand a variety of sample conditions, and, when activated, cleave reporter molecules by “collateral” or trans-cleavage, increasing the detection signal, CRISPR effectors like Cas12, Cas13, and Cas14 are especially well suited for diagnostics [9]. Furthermore, CRISPR/Cas-Dx can distinguish tumor-derived mutant alleles from wild-type backgrounds using guide RNAs (gRNAs) targeting single-nucleotide variations (SNVs), enabling early-stage cancer diagnosis via mutations [10,11]. Since the introduction of Cas-mediated collateral cleavage tests (DETECTR, SHERLOCK), the field has moved quickly. Next-generation designs offer amplification-free detection using highly active Cas enzymes and sophisticated reporters, potentially reducing or eliminating the requirement for polymerase chain reaction (PCR) or recombinase polymerase amplification (RPA), lowering contamination concerns, and simplifying processes [12,13]. However, to quantify absolute single molecules, partition-based digital CRISPR systems employ Poisson statistics in droplets or microcompartments [14]. In addition, CRISPR can be integrated into sequencing workflows, where Cas9-mediated targeted enrichment increases the representation of selected genomic regions before next-generation sequencing (NGS), thereby improving sequencing depth and the detection of low-frequency variants [15,16]. At the same time, portable biosensor platforms are incorporating CRISPR. For example, new technologies combine gold nanoparticle nanoarrays and CRISPR-Cas effectors to isolate tumor-derived EVs and identify exosomal miRNAs with great sensitivity [17]. Rapid point-of-care (POC) cancer screening has been made possible by the application of paper-based and microfluidic CRISPR devices for urinary biomarker detection [18,19]. These advancements show a fundamental change: CRISPR/Cas-Dx is evolving beyond a conventional molecular assay toward a compact, integrated, and potentially field-deployable diagnostic platform. Artificial intelligence (AI) is increasingly being incorporated into CRISPR/Cas-Dx research to improve detection accuracy and decipher complex readouts. Machine-learning algorithms are increasingly being used to optimize gRNA design and predict target specificity, while AI-based approaches may also facilitate signal interpretation and the integration of multidimensional biomarker data into clinical risk models [20,21,22]. The combination of CRISPR/Cas-Dx with AI opens the door to real-time, autonomous diagnostics that can function in settings with limited resources or at the point of care [21,22]. The unique contribution of this review is to connect previously separated areas by providing an end-to-end, translation-focused roadmap for CRISPR/Cas-Dx in cancer detection, from CRISPR recognition and signal generation to multi-omics biomarker detection, biosensor and microfluidic integration, AI-assisted interpretation, and practical barriers to regulatory approval and real-world implementation. Despite the rapid progress of CRISPR/Cas-Dx in cancer research, the existing literature remains fragmented, with studies focusing separately on Cas effector mechanisms, individual biomarker classes, biosensor engineering, AI-enabled analysis, and clinical translation [21,23,24,25]. Most recent reviews focus on one or two of these dimensions, such as specific Cas systems, liquid biopsy biomarker detection, or assay development, but do not integrate them into a single framework that explains how mechanistic advances are being converted into clinically deployable diagnostic platforms. In doing so, this review clarifies how CRISPR/Cas-Dx can move beyond proof-of-concept assays toward scalable, POC, and clinically actionable liquid biopsy tools for early cancer detection and monitoring.

## 2. Evolution of CRISPR/Cas Technology in Diagnostic Milestones

**Figure 1:**
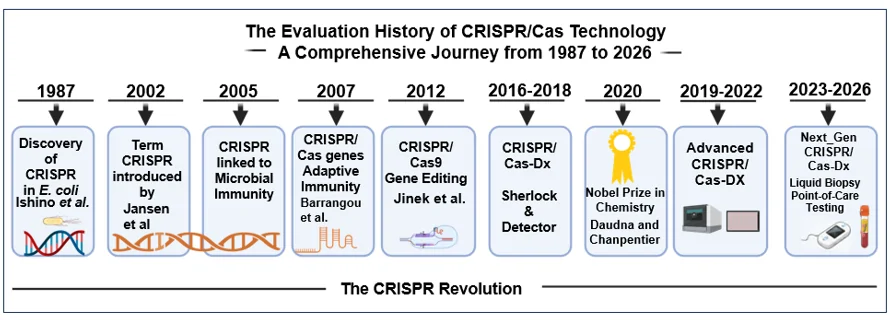
The evolution history of CRISPR/Cas technology based on diagnostic milestones. Milestones are based on Ishino et al. [26], Jansen et al. [27], Barrangou et al. [30], and Jinek et al. [31]. Abbreviations: Cas, CRISPR-associated protein; CRISPR, clustered regularly interspaced short palindromic repeats; CRISPR/Cas-Dx, CRISPR/Cas diagnostics; Next_Gen, next generation.

CRISPR/Cas-Dx have evolved remarkably since the discovery of unusual DNA repeats in *Escherichia coli* by Ishino et al. in 1987 [26]. In 2002, Jansen et al. introduced the term “clustered regularly interspaced short palindromic repeats (CRISPR)” and identified CRISPR-associated (*Cas*) genes [27]. Subsequent studies in 2005 showed that CRISPR spacer sequences were derived from foreign genetic elements, supporting the role of CRISPR systems in microbial adaptive immunity [27,28,29]. In 2007, Barrangou et al. experimentally demonstrated that CRISPR provides acquired resistance against viruses in prokaryotes [30]. The breakthrough came in 2012 when Jinek et al. demonstrated that CRISPR-Cas9 could be programmed for sequence-specific DNA cleavage [31], followed by the adaptation of CRISPR-Cas9 for mammalian cells in 2013, which established the foundation for modern genome engineering [31,32,33]. The development of CRISPR/Cas-Dx was enabled by the discovery and characterization of Cas effectors with collateral nuclease activity. In 2017, the Cas13a-based SHERLOCK platform exploited collateral RNA cleavage for highly sensitive nucleic acid detection. In 2018, the target-activated collateral single-stranded DNase activity of Cas12a provided the mechanistic basis for Cas12a-based diagnostic platforms, including DETECTR [34,35]. Between 2019 and 2022, CRISPR-based diagnostic platforms were expanded through amplification-free detection, electrochemical sensing, microfluidic integration, and multiplexing strategies [36,37,38]. Advances from 2023 to 2026 have further integrated CRISPR/Cas systems with nanomaterial-enabled biosensors, electrochemical and microfluidic platforms, and computational or machine-learning-assisted analytical strategies to improve the sensitivity, quantitative capability, multiplexing potential, and POC applicability of cancer diagnostics [39,40,41,42]. For liquid-biopsy analytes, including cfDNA, ctDNA, exosomal RNA, and miRNAs, CRISPR/Cas-Dx platforms have enabled sensitive and quantitative detection through digital, multiplexed, and amplification-free approaches, with potential applications in early cancer detection [41,42,43,44,45]. Additionally, engineering and optimization of Cas effectors can enhance enzyme activity, target recognition, and analytical specificity, thereby improving the performance and expanding the design flexibility of CRISPR/Cas-Dx platforms [46]. CRISPR/Cas systems have emerged as powerful molecular diagnostic platforms because they enable rapid, programmable, portable, and highly sensitive detection of DNA and RNA biomarkers, while also supporting the indirect detection of protein biomarkers through target-conversion strategies [12,47,48]. A timeline illustrating key diagnostic milestones in the evolution of CRISPR/Cas technology from its initial development to CRISPR/Cas-Dx and precision liquid biopsy applications (2023–2026)) is shown in Figure 1.

## 3. Mechanisms, Classification, and Diagnostic Development of CRISPR/Cas System

The CRISPR/Cas system is one of the most transformative discoveries in molecular biology, having evolved from a microbial adaptive immune system into a versatile platform for genome engineering, molecular diagnostics, and therapeutics. The molecular mechanism underlying CRISPR/Cas immunity consists of three sequential stages: adaptation, expression, and interference [49]. The CRISPR locus is transcribed into a lengthy precursor CRISPR RNA (pre-crRNA) during the expression phase. This pre-crRNA is then processed into short CRISPR RNAs (crRNAs) with spacer sequences. These crRNAs create surveillance complexes by joining forces with Cas effector proteins [50,51]. During the interference phase, the Cas complex is guided by the crRNA to complementary target sequences (protospacers) in the invading DNA or RNA, which results in sequence-specific cleavage and the invader’s demise [50,51,52]. Protospacer adjacent motif (PAM), a brief DNA sequence adjacent to the target site, is essential for differentiating self from non-self-DNA, since it is present in invasive genetic material but absent in the bacterial genome [31].

CRISPR–Cas systems are hierarchically classified according to the architecture of their effector machinery. At the highest level, systems are divided into two classes, defined by whether target recognition and cleavage are mediated by a multi-subunit effector complex (Class 1) or a single multidomain effector protein (Class 2). Within each class, types are distinguished based on signature Cas proteins, evolutionary relationships, and mechanistic features. Recent comprehensive surveys of CRISPR–Cas loci now recognize seven types (I–VII) and at least 46 subtypes across the two classes [53,54]. Multi-subunit effector complexes made up of multiple Cas proteins are used by Class 1 systems (Types I, III, IV and VII). For example, Type I systems use the Cascade (CRISPR-associated complex for antiviral defense) complex for target recognition and the Cas3 helicase–nuclease for DNA degradation. Type III systems, including CRISPR system type III- multi-subunit complex (Csm) and CRISPR module RAMP complex (Cmr), offer adaptable defense by targeting both DNA and RNA [55]. Type IV systems are typically plasmid-associated and encode diverse Class 1 ribonucleoprotein effector complexes, commonly comprising Cas5, Cas7, and subtype-specific accessory proteins, which contribute to plasmid competition and defense against mobile genetic elements [54,56]. Recently proposed Type VII systems constitute a newly recognized Class 1 lineage that assembles Cas5–Cas7 ribonucleoprotein complexes and employs a Cas14-family nuclease for RNA-guided target cleavage, representing a distinct evolutionary branch of Class 1 CRISPR-Cas immunity [57,58].

Single, massive, multidomain effector proteins that mediate both recognition and cleavage are characteristic of Class 2 systems (Types II, V, and VI). Type II systems make use of Cas9, which introduces double-stranded breaks (DSBs) at target locations next to a PAM sequence under the guidance of a crRNA and a trans-activating crRNA (tracrRNA) [31]. Cas12 (Cpf1), which detects T-rich PAMs and produces staggered DNA cuts, is used by Type V systems, increasing its usefulness for certain genome-editing applications [59]. Cas13, which specifically targets RNA and exhibits collateral RNA cleavage activity following target recognition, belongs to the Type VI CRISPR systems [54]. This property has been harnessed in CRISPR/Cas-based nucleic-acid detection platforms, including SHERLOCK, enabling rapid, sensitive, and programmable molecular diagnostics [34].

**Figure 2:**
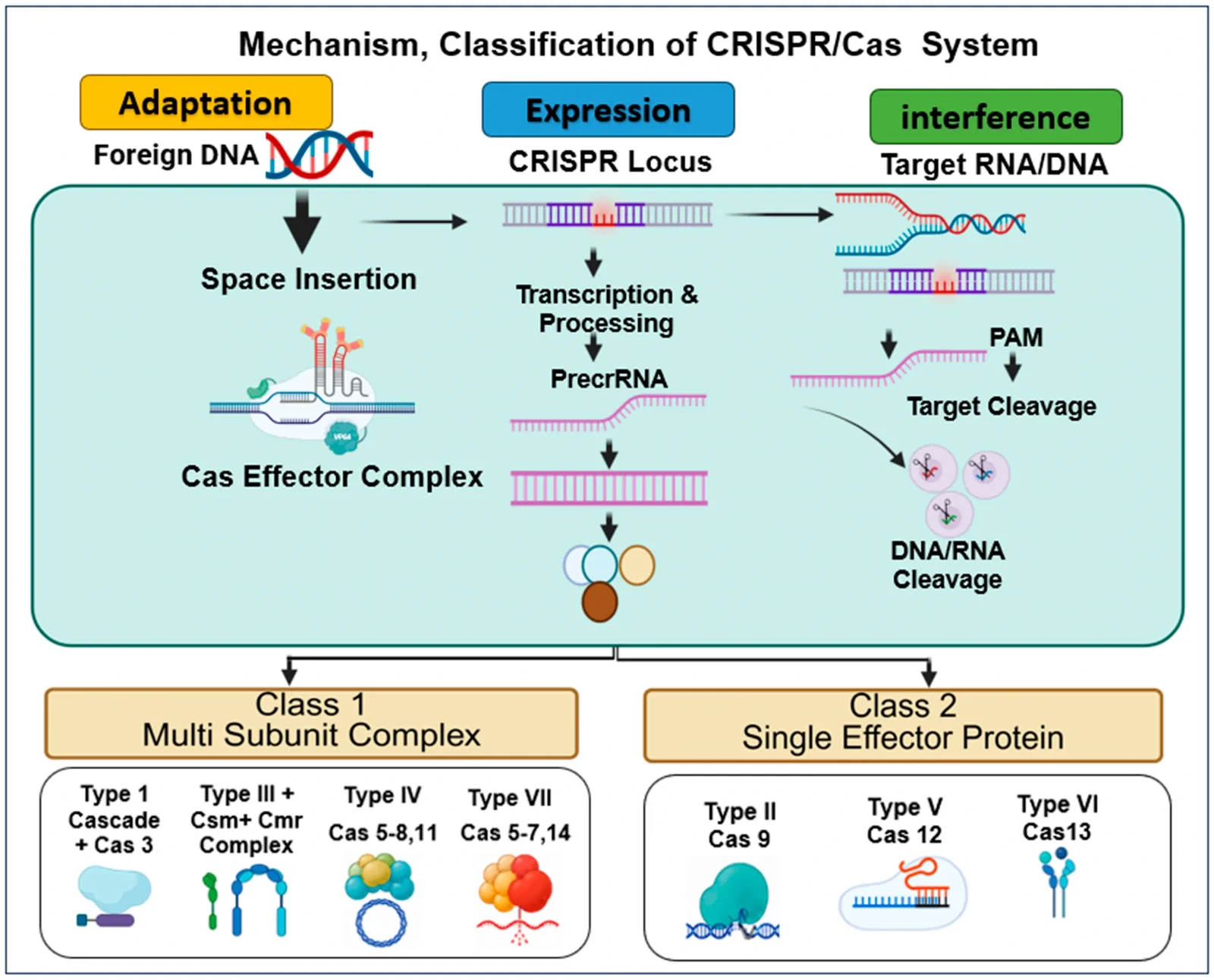
Mechanisms and classification of CRISPR/Cas systems. Schematic illustration of CRISPR/Cas immunity showing the three main stages: adaptation (acquisition of foreign DNA spacers), expression (transcription and processing of pre-crRNA into mature crRNAs), and interference (crRNA-guided recognition and cleavage of target DNA or RNA in a PAM-dependent manner). The lower panel summarizes CRISPR/Cas classification into Class 1 systems (Types I, III, IV, and VII), which use multi-subunit effector complexes, and Class 2 systems (Types II, V, and VI), which rely on single effector proteins such as Cas9, Cas12, and Cas13, etc. Abbreviations: Cas, CRISPR-associated protein; Cmr, CRISPR module RAMP complex; CRISPR, clustered regularly interspaced short palindromic repeats; crRNA, CRISPR RNA; Csm, CRISPR system type III multi-subunit complex; PAM, protospacer adjacent motif; pre-crRNA, precursor CRISPR RNA.

In 2017, the carcinogenic EGFR L858R and BRAF V600E mutations were detected using SHERLOCK technology, marking the first application of CRISPR/Cas-Dx in cancer diagnostics [34]. Additionally, CRISPR/Cas-Dx has advanced into liquid biopsy applications, which offer a noninvasive way to detect and track cancer by detecting ctDNA, miRNAs, CTCs, cfRNA, and tumor EVs [25,60]. Recent developments in CRISPR technology have expanded its uses into single-cell lineage tracing, epigenome editing, transcriptional regulation, and live-cell imaging. Meanwhile, innovations in CRISPR delivery, including lipid nanoparticles, viral vectors, and EVs systems, are tackling issues with clinical translation [61]. Furthermore, the specificity and predictability of CRISPR-based treatments have significantly increased with the use of AI and machine learning (ML) for gRNA design and off-target prediction [22,62]. CRISPR has quickly developed into a fundamental tool that is changing the field of biological and clinical research, from its original discovery in microbial genomes to its current function in precision medicine and diagnostics. An overview of the molecular mechanisms of CRISPR/Cas immunity and the classification of CRISPR/Cas systems is illustrated in Figure 2.

## 4. CRISPR/Cas-Based Diagnostic Platforms for Early Cancer Detection

**Figure 3:**
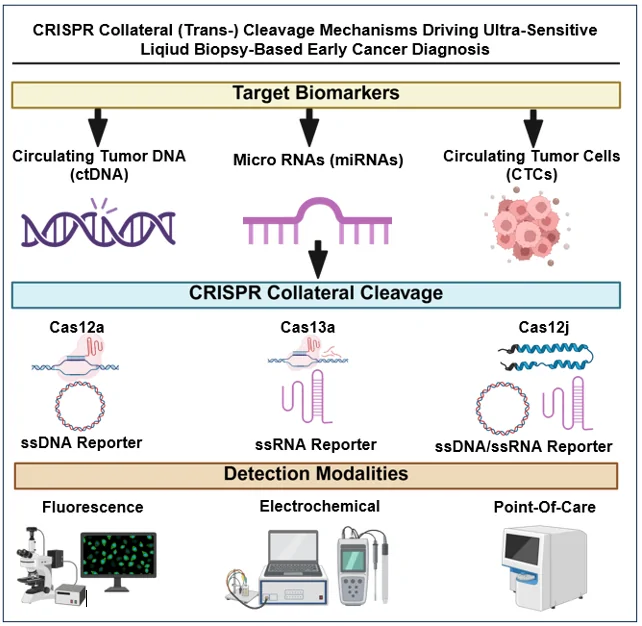
Schematic illustration of CRISPR collateral cleavage mechanisms enabling ultrasensitive liquid biopsy-based cancer diagnosis. Abbreviations: CRISPR, clustered regularly interspaced short palindromic repeats; CTCs, circulating tumor cells; ctDNA, circulating tumor DNA; miRNAs, microRNAs; ssDNA, single-stranded DNA; ssRNA, single-stranded RNA.

**Figure 4:**
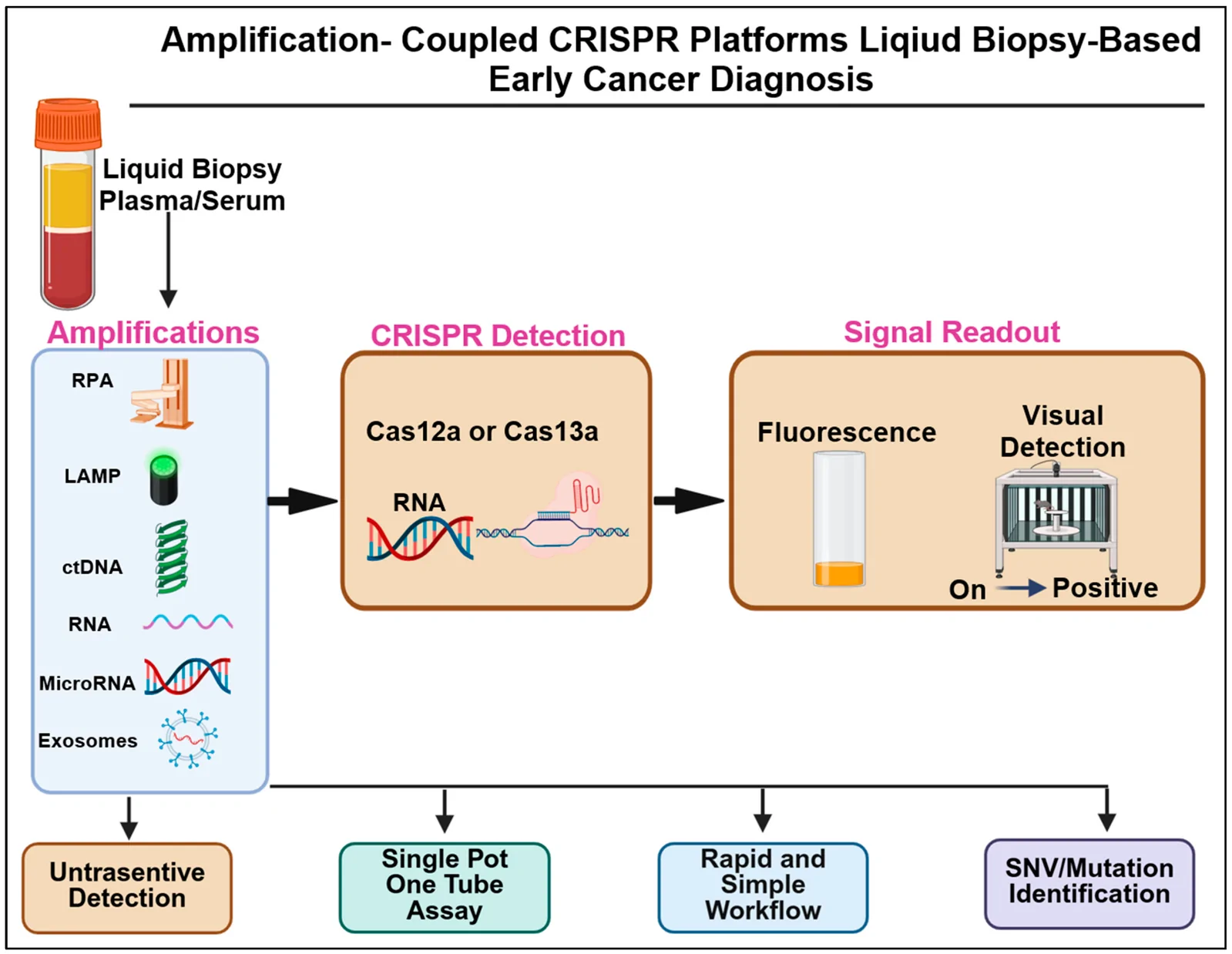
Schematic illustration of amplification-coupled CRISPR/Cas diagnostics integrating isothermal nucleic acid amplification (RPA or LAMP) with CRISPR-based detection. Abbreviations: Cas12a, CRISPR-associated protein 12a; Cas13a, CRISPR-associated protein 13a; CRISPR, clustered regularly interspaced short palindromic repeats; ctDNA, Circulating Tumor DNA; LAMP, loop-mediated isothermal amplification; RPA, recombinase polymerase amplification; SNV, Single Nucleotide Variant.

**Figure 5:**
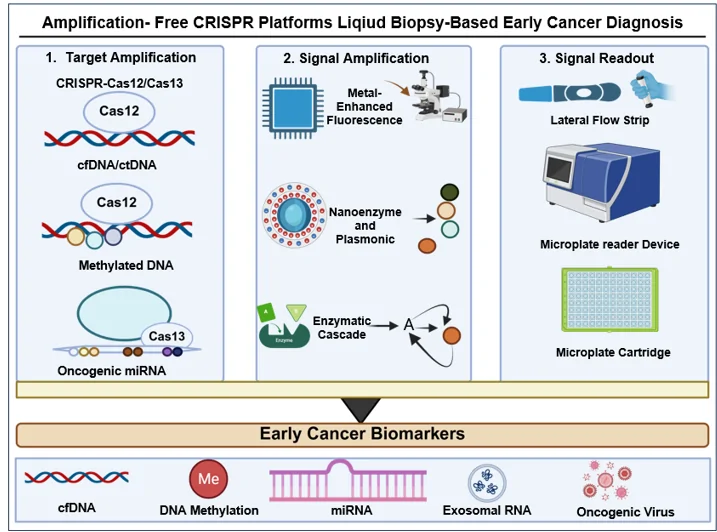
Amplification-free CRISPR/Cas-Dx for liquid biopsy early cancer detection include direct Cas12a/Cas13 target recognition, non-nucleic acid signal amplification, and portable readouts. Abbreviations: Cas12, CRISPR-associated protein 12; Cas13, CRISPR-associated protein 13; cfDNA, cell-free DNA; CRISPR, clustered regularly interspaced short palindromic repeats; miRNA, microRNA.

**Figure 6:**
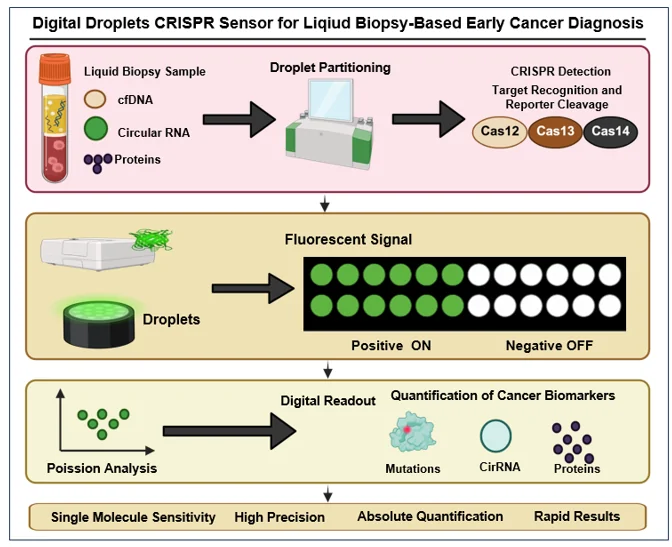
Digital/droplet CRISPR sensors for liquid biopsy-based early cancer diagnosis. Abbreviations: Cas13, CRISPR-associated protein 13; Cas14, CRISPR-associated protein 14; cfDNA, Cell-free DNA; circRNA, circular RNA; CRISPR, clustered regularly interspaced short palindromic repeats.

**Figure 7:**
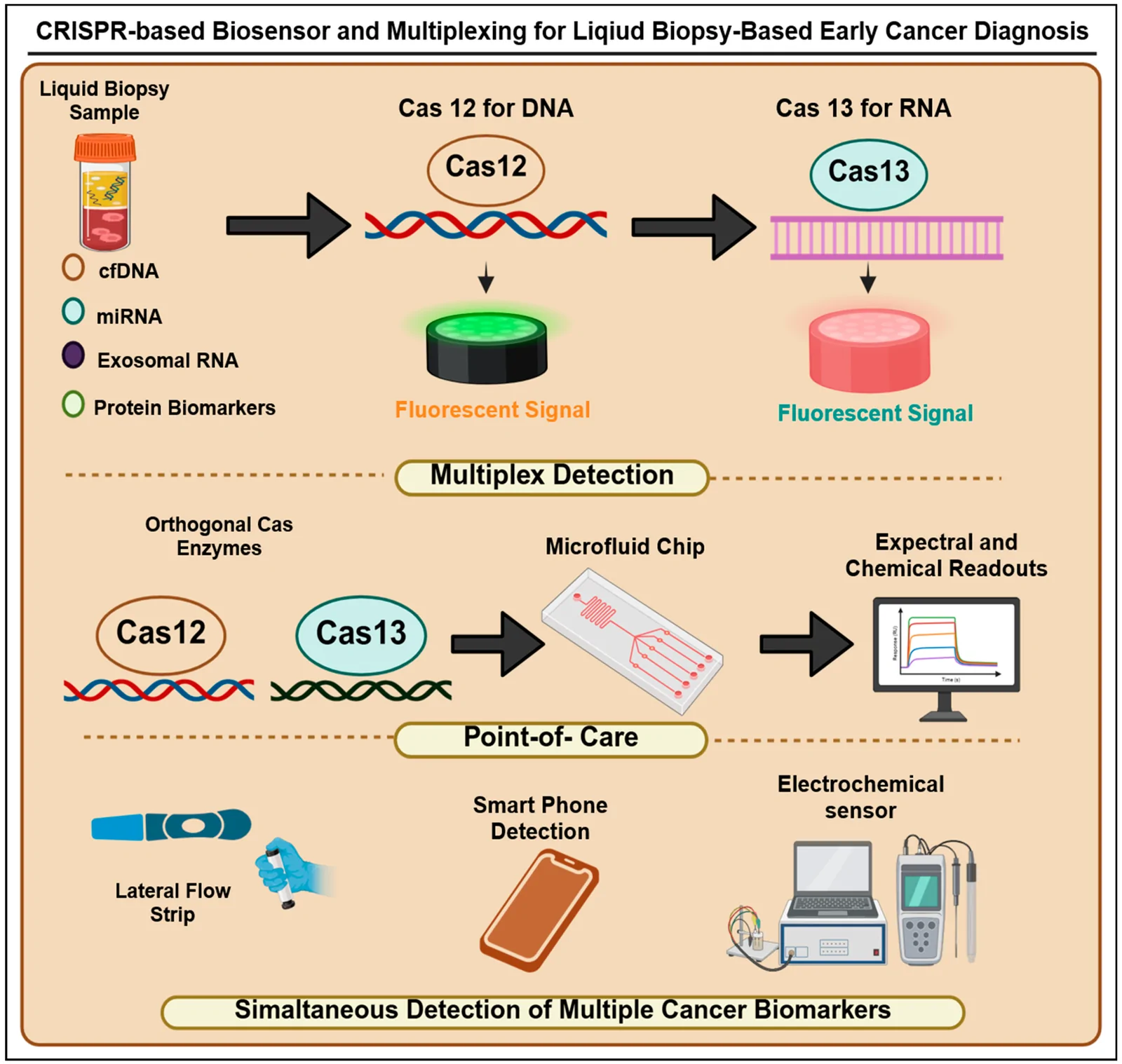
Schematic overview of CRISPR/Cas-Dx biosensors and multiplexing strategies for liquid biopsy-based early cancer diagnosis. Abbreviations: Cas12, CRISPR-associated protein 12; Cas13, CRISPR-associated protein 13; cfDNA, cell-free DNA; CRISPR, clustered regularly interspaced short palindromic repeats; CRISPR/Cas diagnostics; miRNA, microRNA.

CRISPR/Cas-Dx exploit the sequence-specific recognition of CRISPR/Cas systems to detect liquid biopsy biomarkers, including ctDNA, miRNAs, CTCs, EVs, and exosomal RNA, using amplification-free or amplification-assisted strategies with high sensitivity and specificity. Based on their target recognition and signal-generation mechanisms, CRISPR/Cas-Dx can be broadly classified into five major categories: (i) collateral (trans) cleavage-based diagnostic platforms, (ii) amplification-coupled platforms, (iii) amplification-free platforms, (iv) digital or droplet-based platforms, and (v) biosensing and multiplexing platforms (Figure 3, Figure 4, Figure 5, Figure 6 and Figure 7). Representative CRISPR/Cas-based liquid biopsy platforms, together with their analytical performance, validation status, and translational limitations, are summarized in Table 1.

### 4.1. CRISPR/Cas Collateral (Trans-) Cleavage-Based Diagnostic Platforms

One well-established class of liquid biopsy technologies comprises diagnostic tools based on CRISPR/Cas collateral (trans-)cleavage. In these systems, the presence of low-abundance nucleic acid biomarkers is converted into amplified fluorescence, electrochemical, surface enhanced Raman scattering (SERS), or lateral-flow signals when target recognition by guide RNA-programmed nucleases, such as Cas12, Cas13, and Cas14, triggers non-specific catalytic cleavage of reporter molecules [9,86,87,88]. The translational utility of these platforms, notwithstanding their great analytical sensitivity, depends on achieving a balance between sensitivity, specificity, assay turnaround time, sample preparation requirements, multiplexing capacity, workflow complexity, and scalability for clinical deployment. As illustrated schematically in Figure 3, target recognition by guide RNA-programmed CRISPR effectors (Cas12a, Cas13a, and Cas12j) triggers nuclease activation and robust nonspecific trans-cleavage of reporter molecules, resulting in amplified and quantifiable diagnostic signals.

In general, amplification-coupled CRISPR/Cas-Dx systems are more sensitive. For example, the HiCASE platform showed 88.1% sensitivity and 100% specificity in 140 plasma samples from 120 patients with non-small cell lung cancer (NSCLC), detecting EGFR mutations at a variant allele frequency of 0.01% using 40 ng of cfDNA. However, its reliance on restriction enzyme and PCR processes restricts its rapid POC applicability and increases operational complexity [63]. In a similar vein, exponential amplification reaction (EXPAR)-integrated Cas12j (EXP-J) achieved 90% sensitivity and 95% specificity in a lung cancer cohort and allowed femtomolar miRNA detection in about 40 min [64]. Although this method demonstrates the potential of Cas12j for RNA diagnostics, wider clinical translation is limited by its reliance on isothermal amplification and validation in small, single-center cohorts. On the other hand, amplification-free or amplification-minimized approaches reduce the risk of contamination and simplify workflows; however, they often require sophisticated signal amplification strategies or specialized equipment. For instance, a preamplification-free Cas13a-based RNA-Nanocircle autocatalytic system achieved a detection limit as low as 1 aM within 15 min; however, its complex RNA-Nanocircle synthesis and limited stability in untreated plasma remain important limitations [84]. Trade-offs between accessibility and sensitivity are demonstrated by other platforms. By combining exosome collection, isothermal amplification, portable fluorescence detection, smartphone-based analysis, and deep learning, the lectin-induced RPA (LI-RPA)–Cas12a system achieves a visual limit of detection of 100 EVs μL^−1^ and 95% diagnostic accuracy in 100 clinical samples. It still needs exosome-specific preprocessing even if it has great POC potential [65]. With benefits in terms of portability and miniaturization, electrochemical CRISPR biosensors based on Cas12a, Cas13a, and Cas14a allow for the attomolar-to-femtomolar detection of ctDNA, miRNAs, and exosomes. However, industrial standardization and broad acceptance may be hampered by their reliance on synthetic nanomaterials, electrode fabrication, and specialized readout devices. One of these platforms, main advantages is their multiplexing capability: While rolling circle amplification (RCA)–CRISPR techniques allow the simultaneous profiling of several small extracellular vesicle (EV) miRNAs, potentially improving diagnostic specificity in comparison with single analyte assays, split-crRNA Cas12a systems have achieved up to 12-plex miRNA detection [43,45]. Collateral-cleavage CRISPR/Cas-Dx as a whole shows remarkable analytical performance; nonetheless, the most sensitive systems frequently require multistep amplification, while clinically deployable formats prioritize streamlined procedures, portable detection, and automated data interpretation. Fully integrated sample-to-answer systems, standardized multicenter validation, robust multiplexing, contamination control, scalable manufacturing, and analytical frameworks that are clinically interpretable analytical frameworks should be prioritized in future development. Therefore, rather than being defined only by ultralow detection limits, these technologies should be assessed within a multidimensional translational context that balances analytical performance with practical feasibility and clinical suitability.

### 4.2. Amplification-Coupled CRISPR/Cas-Based Diagnostic Platforms

Amplification-coupled CRISPR/Cas platforms represent the most mature and analytically sensitive category of CRISPR/Cas-Dx for liquid biopsy-based early cancer detection. These systems combine nucleic acid amplification methods, such as recombinase RPA, loop-mediated isothermal amplification (LAMP), or nicking enzyme amplification reaction (NEAR), with sequence-specific CRISPR/Cas recognition to enable the detection of extremely low concentrated cancer biomarkers, including ctDNA, miRNAs, RNA fusion transcripts, and exosomal nucleic acids (Figure 4) [60,89]. Following target amplification, guide RNA-programmed Cas effectors—predominantly Cas12a and Cas13a, but also Cas9 and Cas12b—recognize target sequences and activate collateral cleavage of labeled reporter molecules, producing fluorescent, colorimetric, or electrochemical signals with exceptional analytical sensitivity [90,91,92,93,94]. In practical terms, this design offers the best balance between sensitivity and turnaround time among currently available CRISPR/Cas-Dx formats, which is why these systems are especially attractive for liquid biopsy-based cancer screening.

The capacity of amplification-coupled CRISPR/Cas-Dx to identify uncommon mutations at incredibly low allele frequencies while retaining superior single-nucleotide specificity is one of their key advantages. These platforms frequently achieve attomolar sensitivity and reliable discrimination of SNVs by enriching target molecules before CRISPR-mediated detection. This makes them especially useful for early-stage cancer detection where tumor-derived nucleic acids are scarce. Furthermore, compared to traditional PCR-based assays, the majority of assays function at iso-thermal settings and may be incorporated into closed-tube workflows, lowering the risk of contamination while cutting test time [93,94].

Current research demonstrates this strategy’s adaptability to a variety of cancer biomarkers. T-Cas12a/EXPAR-DG4 achieves label-free detection of blood ctDNA with a detection limit of 57 aM for ctDNA analysis by combining exponential amplification with Cas12a-mediated collateral cleavage [95]. Analytical performance comparable to qPCR for NSCLC mutations is demonstrated by the PAM-independent PRC-Cas technology, which uses optimized crRNA design to improve mutant discriminating while lowering false-positive signals with a thoughtfully streamlined methodology [96]. Amplification-coupled systems have been effectively modified for exosomal and RNA biomarkers in addition to ctDNA. For instance, the one-pot LI-RPA-CRISPR/Cas12a test uses deep learning and smartphone-assisted fluorescence analysis to enable sensitive identification of α-fucose-rich tumor-derived exosomes for colorectal cancer diagnosis [65]. Dynamic DNA assembly-assisted CRISPR/Cas12a has further expanded applications to ultrasensitive quantification of circulating miR-21 with femtomolar sensitivity [97], whereas multiplex RT-RPA–CRISPR/Cas12a assays allow simultaneous identification of multiple lung cancer fusion genes within approximately 30 min [66]. Similarly, the Cas12b-based SPEAR platform combines CRISPR detection with NEAR amplification to achieve single-molecule sensitivity and precise SNP discrimination at mutant allele frequencies as low as 0.1%, approaching the analytical performance of sequencing-based methods while significantly reducing assay time [67].

Despite their high analytical sensitivity, amplification-coupled CRISPR/Cas-Dx systems face several barriers to routine clinical implementation, including workflow complexity, amplification-associated bias, challenges in reagent optimization, risks of carryover contamination, and limited multiplexing capacity. Nonetheless, these systems currently achieve the highest sensitivity among CRISPR/Cas-Dx approaches and are particularly well-suited for the detection of ultra-low-abundance biomarkers, such as ctDNA and rare oncogenic mutations. Their high specificity, combined with strong compatibility with liquid biopsy applications, underscores their potential in precision oncology and early cancer detection. However, broader clinical translation will require significant advances in workflow simplification, automated sample-to-answer integration, and rigorous, standardized clinical validation.

### 4.3. Amplification-Free CRISPR/Cas-Based Diagnostic Platforms

For liquid biopsy-based early cancer detection, amplification-free CRISPR/Cas-Dx platforms have emerged as attractive alternatives to amplification-dependent assays because they eliminate the need for PCR or isothermal nucleic acid amplification, thereby reducing workflow complexity, contamination risk, and assay turnaround time. To reach clinically relevant analytical sensitivity, these platforms rely on highly efficient CRISPR-mediated target recognition in conjunction with tailored signal enhancement techniques rather than target amplification [98]. As illustrated in Figure 5, amplification-free CRISPR/Cas-Dx are generally based on three fundamental principles: (i) direct recognition of tumor-derived DNA, RNA, or epigenetic biomarkers by Cas12a or Cas13 effectors; (ii) signal amplification through optical, electrochemical, plasmonic, nanozyme, enzymatic cascade, or autocatalytic mechanisms; and (iii) integration into simplified POC formats such as lateral-flow strips, cartridges, portable biosensors, or microfluidic devices [99]. The fundamental benefit of amplification-free CRISPR/Cas-Dx is their outstanding analytical specificity combined with a streamlined workflow. These assays are especially appealing for decentralized and POC testing because they minimize operator-dependent variability and significantly reduce false positive results due to carry-over contamination by omitting nucleic acid amplification.

Through creative signal-enhancement techniques, recent technical advancements have greatly increased analytical performance. Nanozyme-assisted platforms offer instrument-free visual detection while maintaining excellent analytical sensitivity in contrast to fluorescence-based CRISPR experiments. For instance, Luo et al. developed a Pt@Au nanozyme-mediated CRISPR/Cas12a assay for miR-21 detection that demonstrated its applicability to rapid POC cancer screening by achieving a 0.5 pM detection limit, validating performance in 40 clinical serum samples, and enabling naked-eye diagnosis of hepatocellular carcinoma [100]. In contrast, plasmonic and alkaline phosphatase coupled platforms mainly improve multiplexing capability and analytical specificity for HPV genotyping comparable to PCR-based assays [101]. More advanced amplification-free systems use DNA substrate-mediated autocatalytic circuits to continually regenerate CRISPR activators, allowing for single-step target detection without the need for traditional nucleic acid amplification [102]. Additionally, amplification-free methods have shown outstanding adaptability to a variety of liquid biopsy biomarkers. Fluorescence-coupled amplification-free CRISPR systems (FCAS) and 3D-printed Cas12a devices enable in situ tumor imaging while achieving femtomolar sensitivity for oncogenic miRNAs like miR-10b [103,104]. Similarly, by enabling direct, bisulfite-free detection of plasma cfDNA methylation through methylation-dependent modulation of Cas12a trans-cleavage activity, CRISPR-Methylated DNA Detection Test (CRISPR-MeDNA Test) offers a major leap in amplification-free epigenetic diagnostics. This method improves the practical viability of liquid biopsy-based cancer screening by providing a quick, economical, and straightforward methodology for identifying 5-methylcytosine (5mC)-modified cfDNA compared with traditional bisulfite sequencing [105]. By enabling multiplex fluorescence or lateral-flow detection, discriminating mature miRNAs from precursor molecules, and allowing sensitive quantification of circulating miRNAs directly from plasma, recent developments like the split crRNA-based Cas12a platform (SCas12a) further expand the capability of amplification-free CRISPR [43,106]. Concurrently, ctDNA detection with a detection limit of 0.8 fM was made possible by a fluorescence-free CRISPR/Cas12a–terahertz biosensor, which achieved a three-order-of-magnitude improvement in detection limit over traditional terahertz biosensors [41]. Preamplification-free CRISPR/Cas13a lateral-flow assays provide ultrasensitive miRNA-21 detection by combining Cas13a collateral cleavage with dual SERS/colorimetric readouts for rapid (≤10 min), room-temperature detection at 8.96 aM (SERS) or 1 fM (visual) in spiked blood [68].

Despite recent advances, amplification-free CRISPR/Cas-Dx platforms still face challenges in achieving ultrahigh sensitivity and often require sophisticated signal enhancement strategies that can increase assay complexity and cost. Moreover, clinical validation, standardization, interlaboratory reproducibility, and regulatory translation remain limited. Nevertheless, the absence of target amplification can simplify workflows and support rapid, decentralized testing, making these platforms attractive for POC applications. Future development should focus on multiplex, cartridge-based platforms integrating genetic, epigenetic, and extracellular vesicle biomarkers with portable readers and automated analysis to facilitate clinical translation.

### 4.4. Digital/Droplet CRISPR/Cas-Based Diagnostic Platforms

Recent digital and droplet CRISPR/Cas-Dx platforms represent a shift from qualitative detection toward absolute, single-molecule quantification [107], yet their clinical value varies substantially when assessed against key translational parameters. Analytical sensitivity is a key advantage across platforms, with several methods reaching attomolar to zeptomolar limits of detection. For example, dual-digital immunoassay (DDA) outperforms traditional ultrasensitive tests by more than two orders of magnitude by combining digital microfluidics with Cas13a amplification to achieve ~100 zM sensitivity and single-molecule resolution [42]. Similar detection limits of 5 aM and ~10 aM are achieved by Super-RADAR and PddCas13a, respectively, allowing for reliable quantification of circulating miRNAs without preamplification or with minimal amplification steps [69,73]. Further enabling accurate telomerase activity detection at a single-molecule resolution without complicated preamplification, digital Cas12a-based ddHECTelo improves specificity over conventional TRAP tests [108]. However, sensitivity gains are often accompanied by trade-offs in workflow complexity and infrastructure requirements. Specificity and mutation discrimination remain critical limitations, particularly for low variant allele frequency (VAF) detection in ctDNA. While engineered systems such as TIDE-Cas14a achieve high sensitivity (0.01% VAF) through mismatch-tolerant crRNA design and amplification cascades [72], CRISPR-based approaches may still underperform compared with ddPCR in discriminating single-nucleotide variants at very low abundance, as demonstrated for BRAF V600E mutation detection [109]. This demonstrates that analytical specificity and reproducibility continue to be obstacles to clinical acceptance in mutation-sensitive oncology applications, even with similar detection limits. Workflow integration and assay time differ significantly. The majority of droplet-based Cas13a systems (~60 min) and rapid platforms like Super-RADAR (<25 min) are competitive with current nucleic acid testing [69,71,73]. Clinical viability is improved by fully automated “sample-to-answer” methods, such as DDA and integrated ddPCR microdevices, which minimize manual handling and lower the possibility of contamination [42,110]. Nevertheless, a lot of platforms continue to rely on multi-step processes that include RPA, T7 transcription, or droplet formation, which makes standardization difficult and restricts routine implementation. The ability to multiplex is becoming a distinguishing feature of digital CRISPR systems. Tumor heterogeneity evaluation and clinically meaningful diagnostic accuracy (~92%) are made possible by platforms like ddSEE and the DNA walker–Cas13a droplet system, which allow simultaneous protein and nucleic acid profiling at the single extracellular vesicle level [44,111]. Dual-target detection is also demonstrated in Casµchip and PddCas13a systems [69,70], although most platforms remain limited to low order multiplexing. Signal crosstalk and droplet encoding limitations continue to hinder scalability toward higher-order multiplex analyses. Microfluidic-integrated systems that can handle milliliter-scale plasma and produce tens of thousands of partitions are preferred from a clinical translation standpoint because of their scalability and sample-processing capacity. However, a major obstacle to wider use is the reliance on specialized microfluidic chips, droplet generators, and fluorescence detection systems, which raises costs and decreases accessibility. Although they are more practical, platforms with amplification-free processes or simpler droplet formation (like ddHECTelo) may sacrifice throughput or multiplexing capacity.

Overall, digital CRISPR/Cas-Dx platforms demonstrate superior analytical sensitivity, absolute quantification, and emerging multiplexing capabilities, making them highly promising for early cancer detection using liquid biopsy. However, their routine clinical adoption is currently hampered by low specificity for uncommon mutations, workflow complexity, dependency on specialized instrumentation, and minimal scalability. Future research should prioritize integrated, amplification-free, and highly multiplexed systems with standardized procedures to bridge the gap between analytical performance and clinical utility. Figure 6 depicts a schematic illustration of the liquid biopsy-based cancer detection using the digital/droplet CRISPR/Cas-Dx workflow. Cas12/13/14 partitioning and single-molecule detection are combined in digital/droplet CRISPR for liquid biopsies.

### 4.5. CRISPR/Cas-Based Biosensors and Multiplexing Diagnostic Platforms

CRISPR/Cas-Dx biosensors have emerged as a promising class of molecular diagnostic platforms by integrating the programmable target recognition of CRISPR effectors with diverse signal transduction technologies, including electrochemical, optical, SERS, lateral-flow, and microfluidic systems [24,112,113,114]. Unlike conventional nucleic acid assays, these platforms exploit the collateral cleavage activities of Cas12 and Cas13 to amplify detection signals, enabling highly sensitive analysis of a broad range of cancer biomarkers, including ctDNA, cfDNA, miRNAs, long non-coding RNAs, EVs, proteins, and epitranscriptomic modifications. However, the clinical utility of different CRISPR biosensors depends not only on analytical sensitivity but also on assay time, multiplexing capability, workflow complexity, scalability, and suitability for routine clinical implementation. Figure 7 demonstrates that CRISPR/Cas-Dx biosensors enhance early cancer screening, risk assessment, and disease monitoring in both laboratory and POC settings by enabling sensitive, multiplexed detection of liquid biopsy biomarkers without extensive infrastructure.

Among contemporary technologies, electrochemical and SERS-based biosensors have the highest analytical sensitivity, frequently approaching femtomolar or attomolar detection limits with effective signal processing. For example, an iontronic Cas12a bio-sensor incorporating hydrophobized anodic aluminum oxide nanochannels with a clamped hybridization chain reaction detected m6A-modified MALAT1 and HOTAIR long non-coding RNA_S_ (lncRNAs) with an attomolar detection limit (32 aM), while machine learning-assisted analysis achieved 96.7% diagnostic accuracy for cancer classification [39]. A Cas12a-powered liquid crystal aptasensor detected PSA with a detection limit of 0.28 ag mL^−1^ and demonstrated excellent agreement with ELISA in clinical serum samples, indicating that CRISPR amplification can be easily extended beyond nucleic acid targets [115]. These systems have remarkable analytical performance, but their reliance on nanomaterials, specific substrates, or advanced signal processing algorithms may limit repeatability, raise production costs, and impede large scale clinical standardization. In contrast, amplification-free optical CRISPR biosensors prioritize simplified workflows and quick turnaround while retaining clinically relevant sensitivity. A typical Cas12a-based dark-field microscopy apparatus detected BRCA1-derived cfDNA within roughly 40 min at a detection limit of 0.081 fM without nucleic acid amplification, thus minimizing contamination risk and simplifying sample processing [40]. Similarly, lateral-flow and smartphone-assisted biosensors provide portable, user-friendly readouts with minimal instrumentation, making them ideal for decentralized and POC testing. However, these reduced formats typically have lesser multiplexing capacity and quantitative precision than electrochemical or spectroscopic platforms, demonstrating the trade-off between analytical performance and operational simplicity.

One significant advantage of CRISPR/Cas-Dx biosensors is their compatibility with multiplex detection, which is increasingly necessary to address tumor heterogeneity and increase diagnostic accuracy. Recent solutions combine orthogonal Cas enzymes (Cas12a and Cas13a), programmable crRNA arrays, spectrally encoded reporters, and microfluidic compartmentalization to assess numerous biomarker classes from a single specimen [116,117]. For example, a Cas13a-powered SERS aptasensor combining catalytic hairpin assembly accomplished single-particle detection of stomach cancer-derived exosomes from only 2 μL of serum within 60 min and supports multiplex exosome profiling [118]. Similarly, a dual Cas12a/Cas13a fluorescence platform detected BRCA1 cfDNA and miRNA-10b within one hour, but a mass spectrometry-assisted CRISPR platform measured ctDNA and miR-21 at femtomolar sensitivity without nucleic acid amplification [119,120]. These multiplex systems significantly improve molecular coverage over single-target assays and hold particular promise for complete liquid biopsy and longitudinal disease monitoring.

Despite significant advances, several impediments continue to hamper routine clinical implementation. Increased multiplex capacity raises the dangers of signal interference, cross-reactivity, and assay calibration complexity. Furthermore, standardizing the production of nanomaterial-based electrochemical interfaces, SERS substrates, and microfluidic devices remains challenging, restricting reproducibility across laboratories and large-scale manufacturing. While many biosensors show great analytical sensitivity in proof-of-concept investigations, only a few have passed multicenter clinical validation or incorporation into automated, high-throughput diagnostic procedures. Future development should focus on standardized device production, fully integrated sample-to-answer systems, AI-assisted data interpretation, and large prospective clinical studies to ensure analytical robustness and regulatory compliance.

CRISPR/Cas-Dx biosensors are a versatile diagnostic tool that combines ultrahigh sensitivity with increased mobility and multiplexing capacity. However, no single platform currently optimizes all therapeutically significant factors simultaneously. Electrochemical and SERS systems maximize analytical sensitivity, optical and lateral-flow platforms facilitate rapid proof-of-concept deployment, and multiplex microfluidic devices provide the most complete biomarker profiling while requiring more engineering complexity. As a result, future clinical translation will rely not only on improving analytical performance, but also on developing standardized, scalable, and cost-effective procedures suitable for routine precision oncology.

## 5. Comparison of CRISPR/Cas-Dx Platforms with Conventional Cancer Diagnostic Technologies

Compared with conventional cancer diagnostic platforms, CRISPR/Cas-Dx systems exhibit clear advantages in analytical sensitivity, specificity, programmability, and clinical adaptability across multiple classes of liquid biopsy biomarkers, including CTCs, ctDNA, cfDNA, microRNAs, and SNVs. Traditional biosensing approaches such as electrochemical, plasmonic, nanomaterial-assisted, and microfluidic platforms have achieved notable sensitivity improvements but often rely on complex nanostructures, multistep signal amplification strategies, and sophisticated instrumentation. For example, conventional electrochemical and plasmonic CTC detection platforms typically report detection limits in the range of ~8–52 cells/mL, as demonstrated by 2D semiconductor-based electrochemical impedimetric cytosensors (52.24 cells/mL) and DNA nanostructure-assisted electrochemical biosensors (8–10 cells/mL) [121,122,123,124]. Advanced microfluidic and nanozyme-assisted devices have enhanced sensitivity to around 3 cells/mL; however, these advances are frequently accompanied by increased operational complexity and lower clinical scalability [125]. In contrast, CRISPR-Cas-integrated platforms, particularly Cas12a-based electrochemical biosensors, exploit collateral trans-cleavage-mediated signal amplification to achieve superior analytical performance, as demonstrated by detection limits as low as 2 cells/mL using a mild reduction-activated CRISPR/Cas12a system [126]. These results highlight the intrinsic amplification efficiency and streamlined workflows of CRISPR/Cas-Dx technologies relative to conventional CTC detection methods. For nucleic acid-based cancer diagnostics, CRISPR/Cas-Dx platforms consistently outperform conventional fluorescence, electrochemical, and isothermal amplification assays in sensitivity, mutation discrimination, and assay simplicity. Fluorescence-based CRISPR/Cas12a systems with PAM-independent target recognition and optimized crRNA designs have achieved ultralow limits of detection down to 100 aM for ctDNA mutation analysis in NSCLC with wide linear ranges spanning 100 aM–100 fM [96]. Although standard isothermal amplification techniques like strand displacement amplification and hybridization probe-based assays can achieve comparable sensitivity (LOD = 95 aM), they often lack intrinsic sequence-specific cleavage and require sophisticated probe engineering [127]. Similarly, electrochemical biosensors based on hollow polymeric nanospheres or multi-enzyme amplification techniques offer femtomolar-level detection, but require intricate electrode modification and multistep reactions, which may limit routine clinical implementation [128]. CRISPR/Cas-Dx technologies are further distinguished by their adaptability in readout modalities—fluorescence, electrochemical, colorimetric, SERS, and dark-field microscopy—while preserving excellent quantitative precision. Notably, the T-Cas12a/EXPAR-DG4 sensing strategy integrates exponential isothermal amplification with Cas12a trans-cleavage and a dimeric G-quadruplex reporter, achieving an expanded dynamic range of 100 aM–10 nM and a label-free detection limit of 57 aM [95], surpassing many conventional amplification-based assays. When benchmarked against gold-standard clinical technologies such as qPCR, dPCR, NGS, and immunoassays, CRISPR/Cas-Dx platforms offer a compelling balance between analytical performance and operational simplicity. Although qPCR and dPCR provide high accuracy, they require thermal cycling, expensive instrumentation, and skilled personnel, limiting their portability and POC applicability, while NGS remains costly, time-consuming, and impractical for routine screening despite its high multiplexing capacity. In contrast, CRISPR/Cas-Dx systems function primarily under isothermal conditions, allow for quick detection (typically within 30–60 min), and approach attomolar sensitivity with mutant allele frequency discrimination as low as 0.01–0.001% [72,129]. Advanced platforms such as CASMART, EasyCatch, and TIDE-Cas14a further demonstrate the power of CRISPR/Cas-Dx for SNV [72,129,130], and rare mutation detection by combining isothermal amplification with programmable nuclease activation, achieving robust clinical validation for oncogenic mutations including EGFR, KRAS, BRCA1, and PIK3CA [96,131,132]. These comparisons highlight that CRISPR/Cas-Dx platforms not only outperform many conventional biosensing and molecular diagnostic technologies in sensitivity and specificity, but also address key translational barriers through simplified workflows, reduced instrumentation requirements, and compatibility with portable and POC formats, positioning CRISPR/Cas-Dx as a transformative next-generation technology for precision oncology diagnostics.

## 6. Multi-Omics Biomarkers for CRISPR/Cas-Dx in Liquid Biopsy-Based Early Cancer Diagnosis

The integration of CRISPR/Cas-Dx with multi-omics biomarkers is transforming liquid biopsy-driven early cancer detection by enabling highly sensitive, specific, and minimally invasive tumor profiling. Recent advances in CRISPR-enabled biosensing have demonstrated remarkable analytical performance across diverse biomarker classes, including tumor-derived exosomes (e.g., limit of detection (LOD)~10 particles/µL via split proximity circuit initiated CRISPR-Cas12a system) [85], ctDNA (ctDNA; 5.4 fM using Cas12a-based amplification) [133], circulating tumor cells (CTCs; 2 cells/mL, 82.72% capture efficiency, quantum dot (QD)-based aptasensor guided by CRISPR/Cas12a) [134], and protein biomarkers such as cervical cancer SCCA and CEA tumor proteins marker (LOD: 10 aM), utilizing CRISPR-Cas12a and Cas13a dual-target detection system [135]. In parallel, CRISPR-mediated interrogation of TME regulators further expands diagnostic capabilities [136,137].

Using CRISPR/Cas-Dx platforms, RNA biomarkers offer an essential dimension to tumor profiling. For oncogenic miRNAs such as miR-21 (overexpressed in lung cancer, NSCLC, and CRC), miR-155 (breast cancer), and miR-203b, miR-4661, miR-219a-2 (overexpressed in liver cancer), Cas12 and Cas13 systems exhibit attomolar to femtomolar sensitivity [64,73,75,84]. A possible multi-omics substrate for CRISPR/Cas-Dx platforms is exosomal biomarkers. Exosomal miRNA biomarkers for breast cancer and HCC, CRISPR/Cas13a provides sensitive, affordable miRNA identification in liquid biopsies [78,79]. ctDNA remains one of the most extensively investigated liquid-biopsy biomarkers for CRISPR/Cas-based early cancer detection, particularly for identifying low-frequency somatic mutations and single-nucleotide variants. Cas13a, Cas12a, Cas12b, Cas14a, and Cas9-based platforms have demonstrated the detection of clinically relevant mutations in EGFR, KRAS, PIK3CA, GNAQ, and other cancer-associated genes, with analytical sensitivities approaching single-molecule, attomolar, or 0.01% variant allele frequency levels. Several tests have been tested on patient plasma or blood, including multiplexed systems capable of detecting mutation panels or combining ctDNA with additional indicators. However, most systems continue to rely on upstream amplification, such as RPA, PCR, EXPAR, RCA, or other multistep procedures, which can increase assay complexity and introduce pre-analytical variability [63,67,72,77,81,82,83].

CRISPR/Cas-aptamer-based biosensors can convert exosomal surface-protein recognition into nucleic acid signals that activate Cas12a or Cas13a collateral cleavage. Such techniques have been used to detect tumor-associated exosomal proteins, including PD-L1, EpCAM, MUC1, and other surface indicators. For example, an aptamer–RPA–TMA–CRISPR/Cas13a assay enabled direct detection of exosomal PD-L1 in serum with a reported limit of detection of 10 particles/mL [138], whereas Cas12a-based aptamer platforms have enabled ultrasensitive detection of tumor-related proteins (EpCAM, CD63, and MUC1) positive exosomes [139]. More recently, orthogonal CRISPR–Cas12a/Cas13a systems have enabled multiplexed profiling of exosomal surface proteins, demonstrating the potential of CRISPR/Cas-Dx for multi-marker liquid-biopsy analysis [140]. Beyond circulating nucleic acids, integration of protein and functional biomarkers could further expand the multi-omics potential of CRISPR/Cas-Dx based on liquid biopsy. For example, a magnetic nanoparticle regulated switching aptamer hybridization chain reaction (HCR) coupled with CRISPR/Cas12a enabled ultrasensitive detection of serum alpha-fetoprotein (AFP), an established hepatocellular carcinoma biomarker, with a detection limit of 0.170 ng mL^−1^ and superior sensitivity to conventional ELISA in clinical samples [141]. Similarly, a target induced cleavage, ligation, and transcription activation cascade linked with CRISPR/Cas13a permitted ultrasensitive detection of Flap endonuclease 1 (FEN1) activity in serum, tissues, and living cells, while also facilitating multiplex detection of additional DNA glycosylases [142]. Furthermore, CRISPR-ELISA has recently established the capability of multiplex plasma cytokine profiling, including IL-6, IL-8, and IL-10, for early lung cancer diagnosis [143]. Together, these studies highlight CRISPR/Cas-Dx’s developing ability to detect heterogeneous biomarker classes, including nucleic acids, proteins, cytokines, and enzyme activity, from liquid biopsy samples. Future integration of these complementary molecular layers with transcriptomic, genomic, extracellular vesicle, and clinical data could lead to more comprehensive multi-omics signatures for early cancer diagnosis and enhanced biological stratification.

## 7. AI-Driven CRISPR/Cas Diagnostics for Precision Cancer Detection

AI is currently a key facilitator of CRISPR/Cas-Dx, supporting assay design, analytical performance, data interpretation, and clinical decision-making. AI streamlines the entire CRISPR/Cas-Dx workflow, from gRNA design and reaction optimization to signal extraction and integration of multimodal clinical data [22,144,145,146]. These characteristics are especially important for liquid-biopsy applications, where tumor-derived nucleic acids and other biomarkers in blood might be difficult to detect due to biological and technical noise. One of the first and most profound AI applications in CRISPR/Cas-Dx is the data-driven design of guide RNAs. Deep-learning and ensemble models trained on large, empirically validated cleavage datasets can predict gRNA efficiency, target accessibility, secondary structure propensity, thermodynamic stability, and off-target activity more accurately than rule-based heuristics. DeepCRISPR, CRISPR-Net, and DeepHF (Deep learning-based High-Fidelity) use sequence context, chromatin/epigenetic characteristics, and cleavage profiles to identify active and specific Cas effectors. These models improve single-nucleotide discrimination and reduce false-positive and false-negative rates, making them crucial for detecting clinically actionable cancer mutations in genes like EGFR, KRAS, TP53, and BRAF [22,144,147,148,149,150]. Machine learning has been used to optimize CRISPR biosensor primer sets, reporter chemicals, and isothermal amplification settings, resulting in shorter empirical optimization cycles and improved sensitivity, specificity, and repeatability. Beyond assay design, AI greatly improves diagnostic signal interpretation by extracting robust features from complicated, noisy CRISPR readouts. CRISPR/Cas-Dx platforms generate a variety of signals, including fluorescence kinetic curves, electrochemical amperometric/voltammetric responses, colorimetric intensity changes, SERS spectra, and lateral-flow strip images. Conventional threshold-based calling generally underperforms in low signal-to-noise settings and with intersample variability. Supervised and deep-learning classifiers, such as support vector machines, random forests, gradient-boosted trees, and convolutional neural networks, can learn nonlinear decision boundaries in high dimensional data, enabling accurate positive/negative classification and improved limits of detection at low biomarker concentrations [145,151,152]. Deep learning-based image analysis automates quantification of fluorescence microscopy fields, smartphone-captured lateral-flow experiments, and microfluidic chip images, decreasing observer bias and enhancing cross-lab consistency [22,145,146,153].

AI also addresses a critical difficulty in precision oncology: the integration of complex, heterogeneous biomarker data. CRISPR/Cas-Dx platforms now assess numerous circulating analytes simultaneously, such as ctDNA mutations, circulating RNA, exosomal nucleic acids, DNA methylation marks, and protein biomarkers. Traditional univariate or low-dimensional statistics are inadequate for modeling interactions between various modalities. Machine learning can combine genetic traits, clinical factors, imaging biomarkers, and demographic data to create composite diagnostic signatures that improve early detection, molecular subtyping, prognosis, and therapy selection. Predictive models trained on large, longitudinal cohorts can uncover biomarker trajectories linked with minimal residual illness, therapeutic resistance, recurrence, and metastatic progression, thereby directing personalized treatment regimens [136,154,155].

The combination of AI, single-cell transcriptomics, and CRISPR functional screening has broadened the potential for mechanism-guided precision oncology [156,157]. AI-powered deep-learning and foundation models used on single-cell RNA sequencing (scRNA-seq) data can infer tumor-specific gene regulatory networks, resolve intratumoral heterogeneity, predict cellular states and drug responses, and identify biologically meaningful gene modules for downstream functional validation [158]. Integrating these computational predictions with pooled CRISPR perturbation screens allows for systematic discovery of gene essentiality and functional cancer dependencies, improving the selection of therapeutic targets [159]. Furthermore, AI-assisted computational frameworks integrating CRISPR screening datasets with single-cell transcriptomics and clinical data identified clinically relevant immune regulators such as PTPN2, TNFAIP3, and SOCS1, improving prediction of patient-specific responses to immune checkpoint therapy and supporting biomarker discovery and personalized treatment strategies [160].

AI is also facilitating the clinical translation of portable CRISPR/Cas-Dx by standardizing workflows, ensuring quality, and providing real-time decision support. Although AI-driven laboratory medicine is still in its early stages, evidence suggests that machine learning can support standardized data interpretation across decentralized sites, continuous assay performance monitoring, anomaly detection, automated quality control, and integration with laboratory information management systems. These capabilities can improve analytical reproducibility, ensure regulatory compliance, reduce operator dependent variability, and boost scalability in POC and resource constrained settings. As CRISPR/Cas-Dx advances, integration with AI-enabled digital health infrastructure is projected to provide dependable clinical deployment, longitudinal performance tracking, and evidence-based decision-making in precision oncology and molecular diagnosis [161,162,163,164].

AI-assisted CRISPR/Cas-Dx is still limited in clinical translation due to limited and heavily curated training datasets, inter-laboratory variability, and platform-specific heterogeneity, all of which hinder model generalizability [151,165]. Furthermore, the low interpretability of many AI models undermines clinical validation, clinician trust, and regulatory approval [166,167]. To improve reliability, scalability, and clinical adoption, this sector will require standardized benchmark datasets, multicenter external validation, explainable AI frameworks, robust quality control procedures, and privacy-preserving approaches such as federated learning [151,167].

## 8. Clinical Translation, Commercialization, and Regulatory Landscape of CRISPR/Cas-Based Liquid Biopsy Diagnostics

CRISPR/Cas-Dx is advancing from proof-of-concept assays to clinically applicable platforms for early cancer detection, molecular stratification, and longitudinal disease monitoring. Their main advantages include single-nucleotide discrimination, attomolar to femtomolar analytical sensitivity, less reliance on centralized instrumentation, and compatibility with ctDNA, ctRNA, exosomal markers, and other biofluid analytes. In particular, CRISPR/Cas-Dx integrated electrochemical, lateral-flow, and microfluidic biosensors provide portable forms with potential POC application [48,86,168]. Despite these advancements, normal clinical application remains hampered by significant translational obstacles. Pre-analytical variability in specimen collection, processing delays, plasma matrix effects, nucleic acid degradation, reagent instability, and variances in data-processing pipelines can all affect test performance and reduce reproducibility. These challenges are particularly critical for low-abundance biomarkers, where minor variations in extraction efficiency, guide RNA design, enzyme activity, buffer composition, or reporter chemistry can have a significant impact on limit of detection, false-positive rates, and quantitative accuracy. As a result, analytical standardization should go beyond target recognition to include standardized protocols for blood collection, plasma isolation, input normalization, assay calibration, internal controls, and performance benchmarking against orthogonal reference methods like qPCR, ddPCR, and NGS [46,48,169].

A feasible translational plan should prioritize multilayer analytical validation before widespread clinical implementation. Core performance measures should include LOD, linearity, accuracy, specificity, interference testing, resilience across operators and laboratories, and stability under planned storage and transport conditions. Assay validation for liquid biopsy applications should consider matrix effects from hemolysis, lipemia, anticoagulants, and freeze–thaw cycles, as well as repeatability across clinically relevant allele fractions and biomarker concentrations. Multi-center research are especially essential because many CRISPR/Cas-Dx technologies function well in controlled laboratory settings but have yet to demonstrate reliable cross-site repeatability under real-world situations [48,170].

Quality control and manufacturing readiness are equally important for translation. Scalable deployment will necessitate GMP-compliant enzyme production, lot-to-lot consistency, a defined shelf life, reagent lyophilization, cartridge stability, and manufacturing techniques that maintain activity during packaging, storage, and distribution. Closed-cartridge formats, fail-safe internal controls, and traceable digital readouts can reduce operator dependence and contamination risk in POC systems while increasing test reliability. However, commercialization will depend on the capacity to scale these platforms at an acceptable cost without compromising analytical performance, particularly for multiplex assays and nanomaterial-enabled sensors, which are sometimes challenging to standardize between batches [46,168].

Regulatory planning should be initiated at the earliest stages of CRISPR/Cas-based liquid biopsy assay development. In the United States, such assays are required to comply with Clinical Laboratory Improvement Amendments (CLIA) regulations governing laboratory testing and to follow U.S. Food and Drug Administration (FDA) regulatory pathways for in vitro diagnostics (IVDs), including 510(k), De Novo, or premarket approval (PMA) routes [171,172]. In the European Union, adherence to Regulation (EU) 2017/746 on in vitro diagnostic medical devices (IVDR) mandates comprehensive demonstration of analytical validity, AI-driven analytical system, clinical performance, and implementation of robust post-market surveillance systems [171,173]. For CRISPR/Cas-based diagnostic platforms that incorporate software-driven data processing or AI-enabled analytical components, regulatory assessment should additionally encompass formal software validation, verification of data integrity, and governance of machine-learning-based decision-support workflows [171,172,173].

Clinical adoption will also be determined by reimbursement and cost-effectiveness. Even technically robust assays may fail to gain widespread adoption if they do not offer substantial clinical utility, workflow efficiency, or economic value when compared to established alternatives such as targeted PCR, ddPCR, or sequencing-based diagnostics. As a result, health-economic evaluation should consider per-test costs, turnaround time, downstream savings from earlier diagnosis, reduction in unnecessary procedures, impact on treatment choices, and the ability to improve minimal residual disease (MRD) surveillance or recurrence prediction. Payers are more likely to fund assays that demonstrate outcome benefit, lower overall care costs, and match established reimbursement frameworks for molecular diagnostics [46,174].

Integration into established clinical workflows is another important factor in acceptance. For laboratory medicine, CRISPR/Cas-Dx platforms should be developed to work within sample-to-answer or sample-to-report processes that can be integrated into existing admissions, quality management, result verification, and electronic health record systems. For decentralized testing, the assay must be simple enough to be used in outpatient clinics or resource limited settings while still producing clinically interpretable, traceable, and auditable data. In practice, the most feasible near-term applications are likely to be targeted uses such as confirmatory mutation testing, therapy monitoring, and MRD assessment rather than broad population screening. These scenarios offer clearer clinical decision points and stronger links to patient management [46,169].

Commercialization is being accelerated by collaborations among academic groups, startups, diagnostics companies, and clinical laboratories, particularly for attomolar sensitive ctDNA panels, exosomal biomarker assays, and paper-based or cartridge-based readouts. Nevertheless, translation to routine use will require consensus standards for assay reporting, reference materials, proficiency testing, external quality assessment, and inter-laboratory comparability. Future success will depend less on further gains in analytical sensitivity alone and more on whether CRISPR/Cas-Dx can demonstrate robust performance, manufacturability, regulatory readiness, clinical utility, and economic value in real-world settings. In this sense, the most important translational milestone is not merely technical feasibility, but integration into a regulated, reimbursable, and workflow-compatible diagnostic ecosystem [146].

## 9. Limitations and Challenges of CRISPR/Cas-Based Cancer Diagnostics

Despite significant progress in analytical sensitivity, programmability, and multiplexing capacity, multiple biological, pre-analytical, technical, and translational constraints continue to hamper the routine clinical adoption of CRISPR/Cas-Dx for cancer detection. The first challenge arises from the intrinsic characteristics of liquid-biopsy analytes. ctDNA and ctRNA are typically present at very low concentrations, are highly fragmented, and exhibit limited stability particularly, in early-stage disease, rendering assay performance highly dependent on specimen integrity and handling. Pre-analytical factors, including blood collection tube type, time to plasma separation, centrifugation conditions, storage temperature, and repeated freeze thaw cycles, can substantially affect nucleic acid stability and introduce variability before CRISPR/Cas-based detection [60,86,175]. In addition, cfDNA extraction efficiency varies widely depending on the isolation platform, input volume, carrier chemistry, and purification methodology; even minimal target loss during extraction can significantly compromise sensitivity when variant allele fractions are exceedingly low [175]. Exosome- and EV-based assays face comparable issues, as enrichment methods such as ultracentrifugation, size-exclusion chromatography, immunoaffinity capture, precipitation, and microfluidic isolation differ in recovery yield, purity, selectivity, and scalability [176,177]. As a result, variations in upstream biomarker separation may obfuscate cross-platform comparisons, contributing to inconsistent clinical efficacy of CRISPR/Cas-Dx [176,177].

Technical specificity also deserves significant consideration. Although CRISPR/Cas systems enable programmable sequence recognition, false-positive signals may arise from nonspecific amplification, carryover contamination, suboptimal guide RNA design, off-target cleavage, background collateral activity, reporter instability, or nonspecific activation within complex biological matrices [98]. These limitations are particularly evident in amplification-coupled and multiplexed assays, where amplification bias, signal interference, and cross-reactivity can compromise quantitative accuracy. In contrast, amplification-free systems lessen contamination risk, but they frequently require sophisticated signal amplification algorithms and may have low sensitivity for identifying ultra-rare targets [98]. Furthermore, test performance may vary between reagent batches due to changes in Cas enzyme activity, crRNA synthesis and stability, polymerase or auxiliary enzyme performance, reporter chemistry, nanomaterial properties, and microfluidic or electrode fabrication [98,177]. Batch-to-batch variability can affect reaction kinetics, background noise, LOD, and overall reproducibility [8,98,177,178].

Interlaboratory repeatability remains a significant obstacle to clinical translation. Variability in sample processing processes, nucleic acid extraction efficiency, reagent composition, instrument calibration, data analysis thresholds, and operator-dependent procedures can cause significant variations in assay outputs, especially around the limit of detection [175,177,179]. The lack of defined protocols, verified reference materials, harmonized controls, proficiency testing frameworks, and external quality assessment programs further restricts cross-study comparability [177,179]. As a result, successful clinical implementation will require standardized pre-analytical and analytical workflows, validated sample-to-result pipelines, robust internal quality controls, GMP-compliant production, stringent lot-release criteria, and multicenter prospective validation against established reference standards [177,179]. Finally, the clinical value of CRISPR/Cas-Dx should be evaluated not only based on analytical sensitivity but also on its ability to deliver reproducible, specific, clinically actionable, and cost-effective performance in real-world healthcare settings [98,152,178,179,180].

## 10. Future Perspectives and Conclusions

CRISPR/Cas-Dx is rapidly moving toward clinically useful applications in cancer detection, molecular stratification, and long-term disease monitoring; yet, its translational maturity remains uneven. Although some CRISPR/Cas-Dx approaches have advanced to clinical evaluation, the majority of cancer-focused platforms including, advanced multiplexed, amplification-free, digital, biosensor-integrated, and multi-omics systems are still in the proof-of-concept, analytical validation, optimization, or early clinical evaluation stages. Thus, promising analytical performance in controlled laboratory conditions should not be confused with clinical validation or readiness for routine diagnostic deployment. Multicenter research, independent validation cohorts, consistent performance assessment, and evaluation in real-world clinical workflows are all necessary for establishing clinical value.

Future development should focus on sample-to-answer procedures, quantitative and multiplexed detection, and the integration of CRISPR/Cas-Dx with multi-omics analysis. Combining genomic, transcriptomic, epigenomic, proteomic, and extracellular vesicle-derived indicators could provide a more complete picture of tumor biology, aiding in early identification, molecular categorization, and longitudinal monitoring. However, successful deployment will necessitate consistent pre-analytical and analytical methods, standardized reporting frameworks, validated computational pipelines, and open performance evaluations.

Standardization and quality assurance will be equally critical in clinical translation. CRISPR/Cas-Dx reporting should clearly state the intended use, specimen type, target biomarker, assay architecture, analytical sensitivity and specificity, LOD, precision, repeatability, decision thresholds, and orthogonal validation techniques. Simultaneously, CRISPR-specific external quality assessment (EQA) and proficiency testing programs should be established to ensure inter-laboratory comparability and independent evaluation of assay performance. International regulatory harmonization, which includes the alignment of analytical and clinical validation standards, quality management systems, reference materials, software assisted interpretation, and post market surveillance, would help to facilitate the global translation of these technologies.

In conclusion, CRISPR/Cas-Dx is shifting from an experimental technology to clinically applicable precision diagnostic platforms; however, the majority of cancer focused applications are still in laboratory research or early clinical validation rather than routine use. The next phase of research should therefore focus on multicenter clinical validation, multiplex multi-omics integration, standardized reporting, external quality assessment, and internationally harmonized regulatory processes. These advancements will be critical for turning CRISPR/Cas-Dx’s significant analytical potential into reliable, clinically relevant, and widely applicable cancer diagnosis.

## Figures and Tables

**Table 1:** Emerging CRISPR/Cas-based advanced platforms/strategies for liquid biopsy-based early cancer diagnosis.

Platform/ Strategy	Cas Type	Target Type	Detection Mode	Analytical Performance	Assay Time	Cancer Type	Clinical Validation	Multiplexing	Technology Readiness	Major Limitation	Commercialization Potential	Key Advantages	Decision-Making Pathway	Reference
HiCASE (High-sensitivity PCR-Cas13a with specific restriction Enzyme detection)	Cas13a	ctDNA (EGFR mutations)	Fluorescence readout	LOD: 0.01%, sensitivity 88.1%, specificity 100%	140–160 min	NSCLC	140 human plasma samples	Single- target	Clinical validation,	Single-center validation; PCR- dependent workflow.	Moderate	Clinically validated ultra-sensitive EGFR ctDNA detection.	For EGFR mutation testing in NSCLC liquid biopsy	[63]
EXP-J	Cas12j	miRNA (miR-21, miR-92a)	Fluorescence readout	LOD: 1 fM, sensitivity 90%, specificity 95%	~40 min	Lung cancer	40 human plasma samples	Dual- target	Clinical validation	EXPAR- dependent workflow; single-center validation	Moderate	Rapid dual-miRNA detection with high sensitivity	For rapid lung cancer miRNA detection	[64]
LI-RPA–CRISPR/Cas12a	Cas12a	Exosome	Smartphone-assisted fluorescence	95% accuracy, LOD: 1.0 × 10^2^ EVs µL^−1^ (visual); 2 × 10^6^–1 × 10^2^ EVs µL^−1^	NR	CRC	100 human plasma samples	Single- target	Clinical validation	Exosome isolation; single-center validation	Moderate	Exosome detection for POC testing	For portable CRC exosome analysis	[65]
CRISPR/Dx Fusion Gene Assay	Cas12a	RNA fusion genes (ROS1 fusions)	Fluorescence readout	5–10 copies µL^−1^	~30 min	Lung cancer	Patient cohort	Multiplex	Clinical validation	Limited fusion targets; requires RT-RPA	Moderate	One-pot multiplex fusion gene detection	One-pot multiplex fusion gene detection	[66]
SPEAR	Cas12b	ctDNA SNPs	Fluorescence readout	Single-molecule sensitivity; 0.1% allele frequency	~30 min	Pancreatic cancer	107 clinical samples, 64 cancer patients samples	Single- target	Clinical validation	NEAR amplification required	Moderate–High	Ultra-sensitive SNP detection with one-pot workflow.	For low-frequency ctDNA mutation analysis	[67]
Surface-enhanced Raman scattering (SERS)/lateral flow assay (LFA)-based CRISPR Biosensor	Cas13a	miRNA (miR-21)	SERS colorimetric lateral-flow readout	LOD: 8.96 aM (SERS); 1 fM (visual)	<45 min	Pan-cancer (miR-21)	Spiked human serum (recovery study); no patient cohort	Single- target	Analytical validation	Specialized SERS instrumentation	Moderate	Amplification-free ultrasensitive miRNA detection	For rapid miRNA detection using lateral-flow platforms	[68]
Split crRNA CRISPR Biosensor	Cas12a	Total 12 miRNAs including miRNA-106b-5p, miRNA-9	Alkaline phosphatase-enhanced AuNP SPR signaling	miRNA-106b-5p (LOD = 7.16 fM) and miRNA-9, (LOD = 3.34 fM).	NR	Breast cancer	30 human plasma samples	12-plex	Clinical validation	Complex crRNA design	High	High-level multiplex miRNA profiling without target amplification	For multiplex miRNA profiling in breast cancer	[43]
PddCas13a	Cas13a	circRNA/miRNA (circHIPK3, miR-21)	Fluorescence readout	LOD: ~10 aM	~60 min	CRC	30 Patient plasma/exosomes)	Dual- target	Clinical validation	Requires droplet digital instrumentation	Moderate	Digital quantification of circRNA and miRNA	For simultaneous RNA biomarker quantification	[69]
Casµchip	Cas13a/Cas13b	circRNAs (circWDR37 and circCK1γ3)	Absolute fluorescence quantification	LOD: 100 copies per reaction	~70 min	Early CRC	60 Patient plasma samples	Dual- target	Clinical validation	Microfluidic chip dependence	Moderate	Absolute dual-target circRNA quantification	For plasma circRNA-based CRC screening	[70]
µDCR	Cas13a	circRNA (circWDR37)	Fluorescence readout	10 copies/mL	15 min (to reach a stable reading result)	CRC	96 Patient plasma samples	Single- target	Clinical validation	RPA-dependent amplification; microfluidic workflow	Moderate	Highly sensitive plasma circRNA detection	For low-abundance circRNA measurement.	[71]
TIDE-Cas14a	Cas14a	ctDNA PIK3CA H1047R mutation	Fluorescence readout	0.01% VAF; aM, 100% sensitivity and specificity	~60 min	Breast cancer	32 human plasma samples	Single- target	Clinical validation	Multi-step RPA–T7 workflow	Moderate	Low-frequency PIK3CA ctDNA.	For detecting rare ctDNA mutations	[72]
Super-RADAR	Cas13a	miRNA (miR-21)	Fluorescence readout	5 aM; high SNR (39.35)	<25 min	NSCLC	12 human plasma samples	Single- target	Clinical validation	Digital SHERLOCK fluorescence detection	Moderate	Rapid RT-free digital miRNA quantification	For fast circulating miRNA analysis.	[73]
Iontronic CRISPR/ Cas12a Biosensor	Cas12a	lncRNA: MALAT1, HOTAIR	Iontronic readout	LOD: 32 aM; Linear range: 10^2^–10^12^ aM, Accuracy 96.7%	NR	Lung cancer	Patient cohort	Dual- target	Clinical validation	Specialized iontronic device	Moderate	Detection of m6A-modified RNA with iontronic sensing.	For epitranscriptomic biomarker analysis	[39]
EXPAR-CRISPR Biosensor	Cas12a	ctDNA (KRAS)	Ratiometric electrochemical readout	LOD: 0.11 fM, Linear range: 0 to 1 nM	2.25 h	Pan-cancer (KRAS mutant)	Complex human serum samples	Single- target	Analytical validation	Cas12a- mediated electrochemical biosensor	Moderate	Reliable ratiometric electrochemical ctDNA detection	For electrochemical ctDNA quantification	[74]
Mutually activated dual-exponential amplification (MADEA) DNA machine	Cas12a	miRNA (miR-155)	Fluorescence readout	LOD: 1.26 fM; Dynamic range: 5 fM–10 nM	NR	Breast cancer	Serum samples	Single- target	Clinical validation	Complex dual- amplification workflow	Moderate	Dual-amplification enhances miRNA sensitivity.	For detecting ultra-low miRNA expression	[75]
CHA-CRISPR Feedback Circuit	Cas12a	miRNA (miR-155)	Fluorescence signal with target regeneration	LOD: 250 aM, Linear range: 250 aM–2.5 nM	120 min	Breast cancer	Serum samples	Single- target	Clinical validation	Complex multi-step amplification	Moderate	Self-sustaining signal amplification with target recycling	For maximum-sensitivity miRNA detection	[76]
Allele-specific (AS)-PCR CRISPR/ Cas12a Sensor	Cas12a	ctDNA (GNAQ Q209P mutation)	Fluorescence detection	zM detection limit, 3% fractional abundance ctDNA detectable	170 min	Uveal melanoma	5 human Plasma samples	Single- target	Clinical validation	PCR- dependent workflow; single mutation target	Moderate	High-specificity hotspot mutation detection	For targeted GNAQ mutation analysis in Uveal melanoma	[77]
Immunomagnetic CRISPR Chip	Cas13a	Exosomal miRNAs (8-plex)	Spatially multiplexed detection	78% efficiency, 55% recovery, AUC = 0.871	1 h	HCC	20 serum samples	8-plex	Clinical validation	Specialized microfluidic/immunomagnetic chip	High	Multiplex exosomal miRNA analysis	For automated multiplex liquid biopsy	[78]
ddSEE	Cas12a/Cas13a	EV protein + EV miRNA	Digital microwell fluorescence counting	Sensitivity: 214 EVs/μL; Diagnostic accuracy: 92%	30 min	Breast cancer	31 human plasma samples	Dual- target	Clinical validation	Specialized digital microwell platform	High	Simultaneous single-EV protein and miRNA detection	For multimodal EV profiling	[44]
Logic-Measurer CRISPR Circuit	Cas13a	Exosomal miRNAs (miR-21, miR-375)	Exo III fluorescence logic output	LOD: 2.1–4.4 fM; detection at 1.4 × 10^2^ particles/mL	NR	Breast cancer	315 human samples	Dual- target	Clinical validation	Complex multienzyme workflow	High	Logic-gated multiplex exosomal miRNA detection	For improving diagnostic specificity using multiple biomarkers.	[79]
Terminal deoxynucleotidyl transferase (TdT)-Cas14a Exosome Biosensor	Cas14a	Tumor- derived exosomes	Electrochemical signal	LOD: 80 particles/mL; Linear range: 6.0 × 10^2^–1.0 × 10^5^ particles/mL	NR	Nasopharyngeal carcinoma (NPC)	20 human plasma samples	Single- target	Clinical validation	Aptamer–TdT–Cas14a electrochemical biosensor	Moderate	Highly sensitive electrochemical exosome detection.	For exosome-based NPC diagnosis.	[80]
Primer exchange reaction (PER)-Cas14a Biosensor	Cas14a	ctDNA (EGFR L858R mutation)	Electrochemical redox signal modulation	LOD: 0.34 fM; Dynamic range: 1 fM–1 μM	NR	NSCLC	5 Human serum samples	Single- target	Clinical validation	PER-dependent amplification	Moderate	Detection of EGFR ctDNA mutations.	For EGFR mutation monitoring in NSCLC	[81]
AND logic-gated integration of CRISPR/Cas9-HCR Platform	Cas9	ctDNA (SNVs)	Fluorescence-based readout	LOD: 1 fM; SNV: 0.1% allele fraction	30 min to 4 h	Pan-cancer (KRAS, EGFR, TP53 SNVs)	Simulated samples in 50% serum; no patient cohort	Single- target	Analytical vali-dation	Multi-step HCR workflow	Moderate	High-specificity SNV discrimination	For precise ctDNA SNV detection.	[82]
PASEA	Cas9	ctDNA (KRAS mutations)	Fluorescence readout	Sensitivity 95.24%	NR	PDAC	153 Patient plasma samples	Single- target	Clinical validation	KRAS-specific; RPA-dependent workflow	Moderate	Clinically validated KRAS mutation detection	For KRAS testing in pancreatic cancer plasma	[83]
RNA-Nanocircle Cas13a Biosensor	Cas13a	miRNA-21	Fluorescence readout	LOD: 1 aM	~15 min	CRC	12 Patient plasma samples	Single- target	Clinical validation	Complex RNA-Nanocircle synthesis and limited stability in untreated plasma.	Low- moderate	Ultra-fast amplification-free RNA detection	For rapid RNA detection in research settings	[84]
RCA-CRISPR	Cas12a	Serum sEV miRNAs (hsa-miR-203b-5p, hsa-miR-4661-5p, hsa-miR-219a-2-3p)	Fluorescence-based multiplex detection	LOD: 3.12 pM; AUC: 0.81	NR	Liver cancer	20 Patient plasma samples	Multi- target	Clinical validation	RCA-dependent amplification	Moderate	Multiplex small EV miRNA detection	For multi-marker liver cancer screening	[45]
Split proximity Circuit (SPC)-CRISPR/Cas12a system	Cas12a	Exosomal surface proteins (PS, MUC1, EpCAM)	Fluorescence readout	LOD: 10 particles/μL (R^2^ = 0.990); AUC: 0.9778, accuracy 91.23%	4–5 h	Breast cancer	57 human serum samples	Multi- target	Clinical validation	Specialized probe design; clinical validation limited to a single cohort	High	Multiplex profiling with high diagnostic accuracy	For early breast cancer liquid biopsy	[85]

Abbreviations: aM, attomolar; AS-PCR, allele-specific polymerase chain reaction; AUC, area under the curve; AuNP, gold nanoparticle; Cas, CRISPR-associated protein; Casµchip, Cas13a/b-based droplet microchip; CHA, catalytic hairpin assembly; CHA-CRISPR, catalytic hairpin assembly with CRISPR/Cas systems; circHIPK3, circular RNA homeodomain-interacting protein kinase 3; circRNA, circular RNA; circWDR37, circular RNA WD repeat-containing protein 37; CRC, colorectal cancer; CRISPR, clustered regularly interspaced short palindromic repeats; ctDNA, circulating tumor DNA; ddSEE, digital dual CRISPR-Cas-powered single extracellular vesicle evaluation; EGFR, epidermal growth factor receptor; EpCAM, epithelial cell adhesion molecule; EVs, extracellular vesicles; EXP-J, exponential amplification reaction with Cas12j; EXPAR, exponential amplification reaction; GNAQ, guanine nucleotide-binding protein G(q) subunit alpha; HCC, hepatocellular carcinoma; HCR, hybridization chain reaction; HiCASE, high-sensitivity PCR-Cas13a with specific restriction enzyme detection; HOTAIR, HOX transcript antisense RNA; LFA, lateral-flow assay; LI-RPA, lectin-induced recombinase polymerase amplification; LOD, limit of detection; lncRNA, long non-coding RNA; MADEA-DNA machine, mutually activated dual-exponential amplification DNA machine; MALAT1, metastasis-associated lung adenocarcinoma transcript 1; miRNA, microRNA; MUC1, mucin 1; µDCR, microfluidic droplet CRISPR/Cas13a; NEAR, nicking enzyme-amplification reaction; NPC, nasopharyngeal carcinoma; NR, not reported; NSCLC, non-small-cell lung cancer; PASEA, programmable enzyme-based selective exponential amplification; PDAC, pancreatic ductal adenocarcinoma; PddCas13a, polydisperse droplet digital CRISPR/Cas13a; PER, primer exchange reaction; PIK3CA, phosphatidylinositol-4,5-bisphosphate 3-kinase catalytic subunit alpha; R^2^, coefficient of determination; RCA, rolling circle amplification; RPA, recombinase polymerase amplification; RT, reverse transcription; SERS, surface-enhanced Raman scattering; SERS/LFA, surface-enhanced Raman scattering/lateral-flow assay; sEV, small extracellular vesicle; SHERLOCK, specific high-sensitivity enzymatic reporter unlocking; SNP, single-nucleotide polymorphism; SNR, signal-to-noise ratio; SNV, single-nucleotide variant; SPC, split proximity circuit; SPEAR, specific point mutation evaluation via CRISPR-Cas assisted recognition; SPR, surface plasmon resonance; Super-RADAR, superfast and reverse transcription-free digital one-pot SHERLOCK; TdT, terminal deoxynucleotidyl transferase; TIDE-Cas14a, T7-integrated duplex enhancer CRISPR/Cas14a; VAF, variant allele frequency; zM, zeptomolar, etc.

## Data Availability

No new data were created or analyzed in this study. Data sharing is not applicable to this article.

## Abbreviations

AFP	Alpha-fetoprotein
AI	Artificial intelligence
aM	Attomolar
AUC	Area under the curve
AuNP	Gold nanoparticles
Cas	CRISPR-associated protein
CEA	Carcinoembryonic antigen
cfDNA	Cell-free DNA
cfRNA	Circulating cell-free RNA
CHA	Catalytic hairpin assembly
circHIPK3	Circular RNA homeodomain-interacting protein kinase 3
circRNA	Circular RNA
circWDR37	Circular RNA WD repeat-containing protein 37
Cmr	CRISPR module RAMP complex
CNN	Convolutional neural network
CRC	Colorectal cancer
CRISPR	Clustered regularly interspaced short palindromic repeats
CRISPR/Cas-Dx	CRISPR/Cas diagnostics
crRNA	CRISPR RNA
Csm	CRISPR system type III multi-subunit complex
CT	Computed tomography
CTC	Circulating tumor cell
ctDNA	Circulating tumor DNA
ctRNA	Circulating tumor RNA
DDA	Dual-digital immunoassay
ddPCR	Droplet digital polymerase chain reaction
ddSEE	Digital dual CRISPR-Cas-powered single extracellular vesicle evaluation
DETECTR	DNA Endonuclease-Targeted CRISPR Trans Reporter
dPCR	Digital polymerase chain reaction
DSB	Double-stranded break
EGFR	Epidermal growth factor receptor
ELISA	Enzyme-linked immunosorbent assay
EpCAM	Epithelial cell adhesion molecule
EQA	External quality assessment
EV	Extracellular vesicle
EXPAR	Exponential amplification reaction
FCAS	Fluorescence-coupled amplification-free CRISPR system
FEN1	Flap endonuclease 1
GNAQ	Guanine nucleotide-binding protein G(q) subunit alpha
gRNA	Guide RNA
HCC	Hepatocellular carcinoma
HCR	Hybridization chain reaction
HOTAIR	HOX transcript antisense RNA
IVDR	In Vitro Diagnostic Regulation
LAMP	Loop-mediated isothermal amplification
LFA	Lateral-flow assay
lncRNA	Long non-coding RNA
LOD	Limit of detection
MALAT1	Metastasis-associated lung adenocarcinoma transcript 1
miRNA	MicroRNA
ML	Machine learning
MRD	Minimal residual disease
MRI	Magnetic resonance imaging
MUC1	Mucin 1
NEAR	Nicking enzyme amplification reaction
NGS	Next-generation sequencing
NPC	Nasopharyngeal carcinoma
NSCLC	Non-small-cell lung cancer
PAM	Protospacer adjacent motif
PASEA	Programmable enzyme-based selective exponential amplification
PCR	Polymerase chain reaction
PDAC	Pancreatic ductal adenocarcinoma
PET	Positron emission tomography
PIK3CA	Phosphatidylinositol-4,5-bisphosphate 3-kinase catalytic subunit alpha
POC	Point-of-care
pre-crRNA	Precursor CRISPR RNA
PSA	Prostate-specific antigen
QD	Quantum dot
qPCR	Quantitative polymerase chain reaction
RCA	Rolling circle amplification
RPA	Recombinase polymerase amplification
SCCA	Squamous cell carcinoma antigen
SERS	Surface-enhanced Raman scattering
sEV	Small extracellular vesicle
SHERLOCK	Specific high-sensitivity enzymatic reporter unlocking
SNP	Single-nucleotide polymorphism
SNR	Signal-to-noise ratio
SNV	Single-nucleotide variant
SPR	Surface plasmon resonance
ssDNA	Single-stranded DNA
ssRNA	Single-stranded RNA
TdT	Terminal deoxynucleotidyl transferase
TMA	Transcription-mediated amplification
TME	Tumor microenvironment
VAF	Variant allele frequency
